# Supplementary figures and images for: A cytidine deaminase regulates axon regeneration by modulating the functions of the Caenorhabditis elegans HGF/plasminogen family protein SVH-1
Source: PLoS Genet. 2024 Jul 26;20(7):e1011367. doi: 10.1371/journal.pgen.1011367 (PMC11305532; doi:10.1371/journal.pgen.1011367)

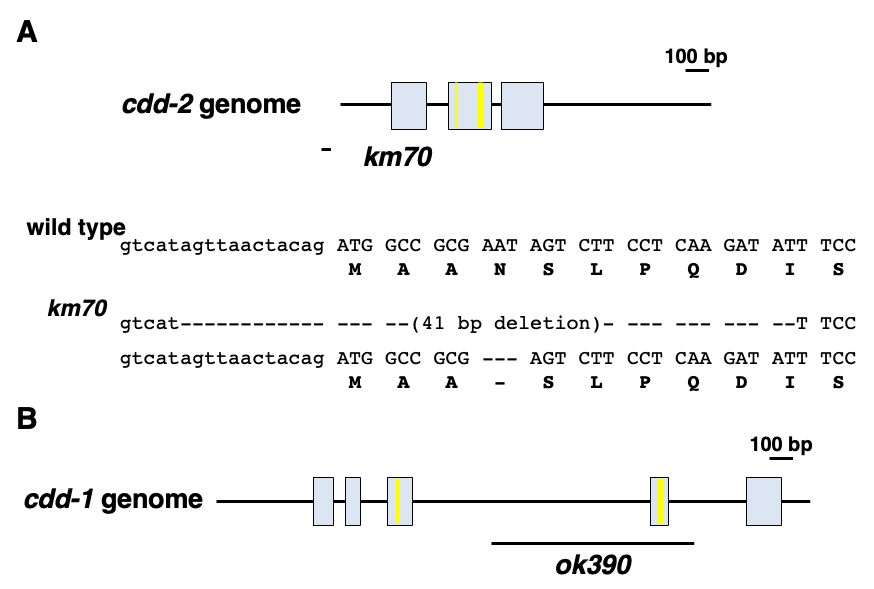

Supplement: S1 Fig — (A and B) Genome structures of the svh-17/cdd-2 (A) and cdd-1 (B) genes. Exons are indicated by boxes, introns and untranslated regions by bars. The domain shown is the zinc-binding region (yellow). The bold line below indicates the extent of the deletion region in the km70 mutant. Nucleotides and corresponding amino acids around the deleted region are also indicated. The cdd-2(km70) mutant contains duplicated DNA regions that include fragments of the cdd-2 gene. One of these regions has a 41 bp deletion that lacks the initiation codon for cdd-2. The other carries a 3 bp in-frame deletion that removes the fourth Asn of CDD-2. The cdd-1(ok390) mutant harbors a deletion of 898 bp and an insertion of 7 bp, removing the zinc-binding region. (TIF) [file pgen.1011367.s001.tif]

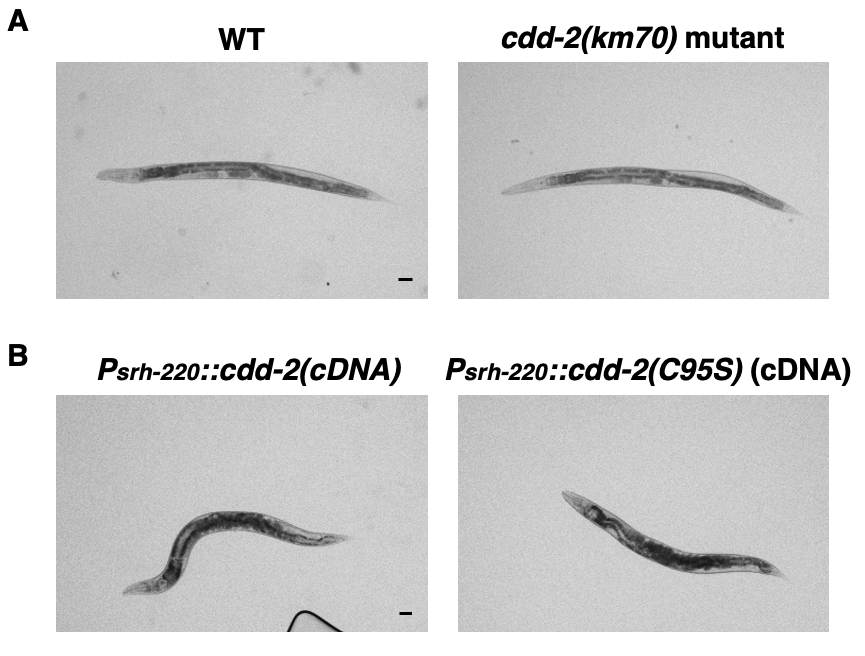

Supplement: S2 Fig — (A) WT and cdd-2(km70) mutant animals grown for 4 days are shown. Scale bar = 50 μm. (B) WT animals carrying Psrh-220::cdd-2 (cDNA) or Psrh-220::cdd-2(C95S) (cDNA) grown for 4 days are shown. Scale bar = 50 μm. (TIF) [file pgen.1011367.s002.tif]

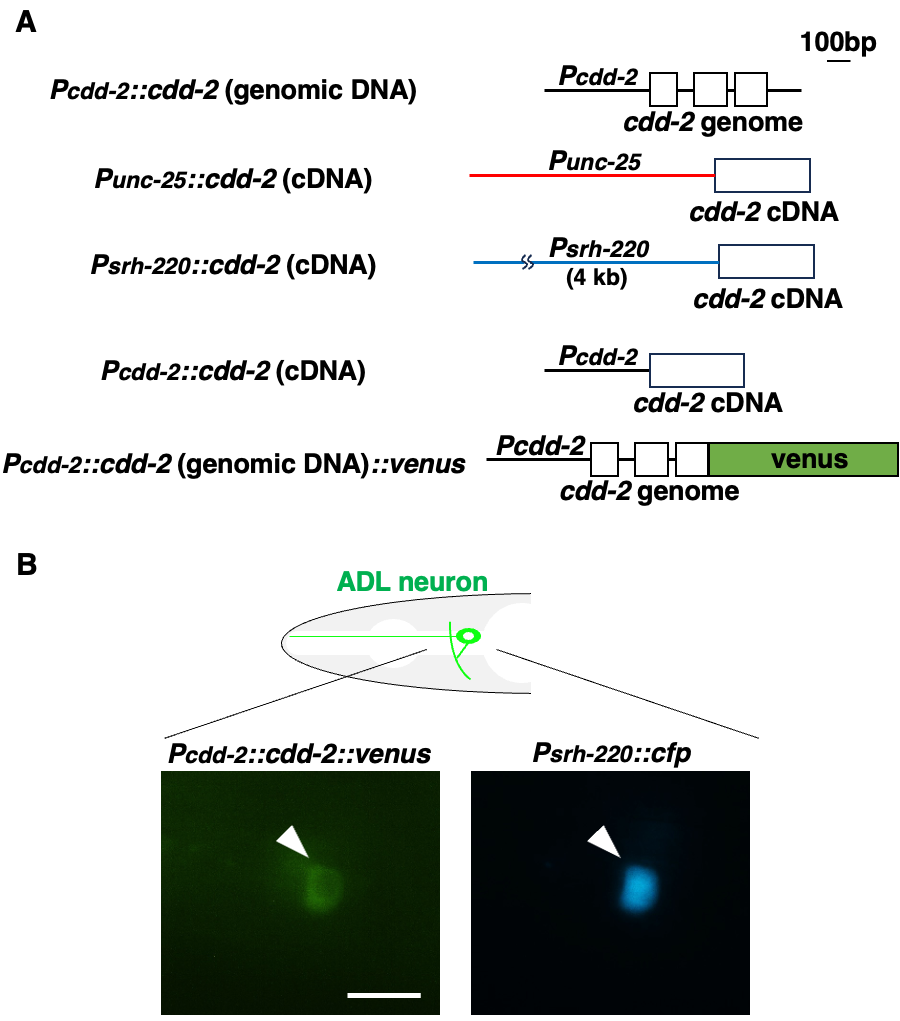

Supplement: S3 Fig — (A) Genomic structures of fusion genes. Exons are indicated by boxes, introns and untranslated regions by bars. (B) Expression of the Pcdd-2::cdd-2 (genomic DNA)::venus gene in ADL neurons. Fluorescent images of animals carrying Pcdd-2::cdd-2 (genomic DNA)::venus at young adult stage are shown. ADL neurons (arrowheads) are visualized by CFP under the control of the srh-220 promoter. Anterior is to the left. Schematic diagram of ADL neurons in the head is shown. Scale bar = 10 μm. (TIF) [file pgen.1011367.s003.tif]

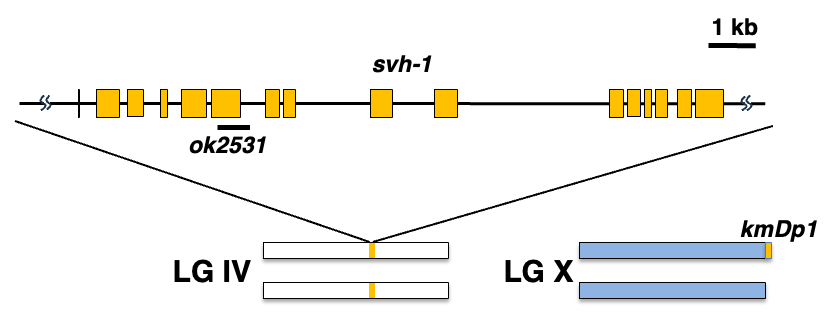

Supplement: S4 Fig — The svh-1(ok2531); kmDp1/+ strain has the svh-1(ok2531) mutation and an extra copy of the svh-1 gene fragment (kmDp1) translocated to the right end of the LGX. (TIF) [file pgen.1011367.s004.tif]
