# Supplementary material for: A cytidine deaminase regulates axon regeneration by modulating the functions of the Caenorhabditis elegans HGF/plasminogen family protein SVH-1
Source: PLoS Genet. 2024 Jul 26;20(7):e1011367. doi: 10.1371/journal.pgen.1011367 (PMC11305532; doi:10.1371/journal.pgen.1011367)
Supplement: Attachment — Submitted filename: response letter-final.docx [file pgen.1011367.s008.docx]

Dear Drs. Hart and Dr. Copenhaver,

Please find enclosed our revised manuscript entitled, “A cytidine deaminase regulates axon regeneration by modulating the functions of the *Caenorhabditis elegans* HGF/plasminogen family protein SVH-1” (by Shimizu et al., PGENETICS-D-23-01297). Thank you for your e-mail of February 10, 2024, and the reviewers’ comments on our manuscript. We appreciate these comments as they have helped us to improve the manuscript. We have conducted further experiments that have been designed to address the comments of the reviewers. We hope this revised manuscript is now acceptable for publication in *PLOS Genetics.* Our point-by-point responses to the reviewers’ comments are provided below.

With best regards,

Kunihiro Matsumoto

Paper: PGENETICS-D-23-01297

Authors: Shimizu et al.,

Title: A cytidine deaminase regulates axon regeneration by modulating the functions of the *Caenorhabditis elegans* HGF/plasminogen family protein SVH-1

The manuscript has been revised according to the comments of the Reviewing Editor and the three reviewers. Our responses to the comments are as follows:

**Responses to the comments of the Reviewing Editor**

**- Increase detail in reporting methods and reagents. This includes a) fully describing the genotype of juIs76 in the first entry row in Table S2 and fully describe the balancer/duplications in this table, b) listing any primers used for plasmid construction and sequence of reagents used for CRIPSR genome editing, and c) concentrations of other plasmids or DNA in microinjection pools used for transgenesis.**

As suggested, we have provided more detailed information on the methods and reagents used in this study.

We have described the genotype of juIs76 and balancer/duplications (S2 Table).

We have listed the oligonucleotide primers used in this study (S3 Table).

We added the information in the Methods section.

**- Comply with the PLOS Genetics Data policy by providing a full data table for experimental results or increasing clarity in data presentation. Does Table S1 contain all experimental results? If so, the reuse of data in different panels/figures within the manuscript needs to be made clear and explicit. Readers expect that independent determinations underlie different panels (like 2C and 5B for WT); if different panels in this manuscript use the same biological replicates/determinations, any data reuse must be explicitly stated in every figure legend, in the Methods (under Statistical analysis), and in the manuscript text.**

As suggested, we have included a complete table of experimental results and improved the clarity of data presentation.

**Our responses to the comments of Reviewer #1**

**1. The main question is somewhat vague. The keywords like protease activity, RNA editing, switching mechanism, and axon regeneration seem equally discussed and in what context the authors try to discover the signaling mechanism is unclear. From the abstract, it seems that the authors are trying to emphasize the finding of the switching mechanism of protease activity, but in what context it is important is not enough explained. Does the switching mechanism of protease activity possibly contribute to the biological process other than the axon regeneration of C. elegans? Or, what is the physiological meaning of the protease activity-based switching mechanism for axon regeneration and larval growth? Given that the RNA editing mechanisms for switching a protein function are already reported, clarifying the difference between the reported studies and the current study in the context of biological importance should be necessary. For example, what is the difference from the following study in terms of the scientific focus?**

As suggested, we have rewritten the Abstract and Introduction.

**2. What triggers the SVH-2? I am wondering whether the inactive form of the protease is enough to activate SVH-2 or not? In other words, is there any specific motif to activate SVH-2, and does the modification by CDD-2 specifically replace the protease active form with the exact motif for activating SVH-2?**

Genetic findings suggest that CDD-2 deaminates the C of *svh-1* mRNA to U, replacing His-755 (CAU) with tyrosine (UAU) in the Asp–His–Ser catalytic triad, resulting in SVH-1, which acts as a growth factor. Thus, the Tyr-755 form of SVH-1 loses protease activity but gains the ability to act as a growth factor. There are two possible explanations for these results. First, the activation of SVH-2 by SVH-1 requires the inactivation of the protease-active SVH-1. Second, the His-755 form of SVH-1 does not function as a growth factor. The *svh-1(S898F)* mutant, which has a Phe-898 residue instead of Ser-898 in the Asp-His-Ser catalytic triad, loses protease activity but fails to suppress the *cdd-2* defect (Fig 4B). Thus, the protease-inactive SVH-1(S898F) requires CDD-2-mediated substitution of His-755 with Tyr-755 in *svh-1* mRNA to function in axon regeneration, suggesting that SVH-1 with His-755 cannot interact with or activate SVH-2. Therefore, CDD-2 specifically changes the His-755 of SVH-1 to a motif that can activate SVH-2.

**3. Does the laser surgery for the L1 larvae inhibit axon regeneration? If the proposed hypothesis is true, L1 larvae should have a different phenotype compared to the adults. Most probably, because L1 has basically active regeneration properties, the laser surgery of L1 still shows active regeneration same as the adults. Still, using overexpression or genetic manipulation can show the different phenotypes of L1 larvae for the laser axotomy**.

Axon regeneration in *C. elegans* is controlled by multiple signaling pathways, such as JNK and p38 MAPK pathways. The SVH-1–SVH-2 signaling pathway regulates axon regeneration at L4 and adult stages, but this pathway may not function in L1 larvae.

**4. ADL neuron is located at the head ganglion while the D neuron is at the body. How is the inactivated SVH-1 at the ADL transported to the D neuron?**

With regard to this comment, we are also interested in the question. SVH-1 in ADL neurons might be transported to D neurons by diffusion or exosomes. However, there is still no experimental evidence for this question.

**5. On line146, the authors conducted the overexpression experiments with the ADL-specific promotor. I am wondering whether the expression of SVH-1 with the intrinsic promotor of SVH-1 suppresses the cdd-2 mutant defect or not. Such experiments clarify the possibility of the involvement of the non-ADL SVH-1 in axon regeneration.**

*svh-1* gene expression is exclusive in ADL neurons (Ref. 5). *svh-1* defects in larval development and axon regeneration are rescued by *svh-1* expression in ADL neurons. Therefore, we have predicted that non-ADL SVH-1 is not involved in axon regeneration.

**6. The way of statistical analysis is very tricky or even awkward. To my knowledge, Fisher’s exact test determines independence or association between two categorical variables. When a contingency table is given, one p-value will be calculated by the test. The authors somehow calculate multiple p-values for Figs 2, 3, 4, and 5 as if Fisher’s test is used for the comparison among the multiple groups to determine whether the groups are statistically different. Although I believe that the way of statistical tests do not affect the conclusion in this case, at least the authors should clarify the precise protocol and the validity for the statistical analysis since the authors unusually use Fisher’s exact test. Also, the issue of multiple comparisons for statistical tests might be involved in this case.**

It is suggested that no adjustment is necessary if each data set is interpreted as resulting from simple parallel-group multi-arm trials of different genotypes with shared control (Ref. 30). Our views have been described in the Methods section.

**7. Most of the evidence for the hypothesis is shown indirectly. Ideally, direct monitoring for the mRNA editing or protease activity will support the proposed hypothesis greatly. Is it possible to detect the deaminated mRNA sequence by RNAseq? Probably the mRNA from different sources such as the intestine interferes with the target processed mRNA, but still, the comparison between cdd-2 mutant and wildtype, or L1 and adult should be different if the proposed hypothesis is true. At least, including the limitation of the current study in the discussion section will clarify the meaning of the current research and provide the basis for future studies.**

We have not yet obtained biochemical evidence for CDD-2 editing of *svh-1* mRNA in *C. elegans*. Detecting *svh-1* mRNA-editing within *C. elegans* would be considerably more difficult if CDD-2 activity is activated by axon injury. In that case, mutants with constitutively active CDD-2 would be required. Therefore, it is necessary to understand the mechanisms that regulate CDD-2 activity.

We have described this point in the Discussion.

**8. Since the beginning of the discussion section starts with the literature/hypothesis review and the result of the current research starts from the second paragraph, the structure of the discussion part looks unbalanced. Probably the current second paragraph starting from line 271 should be moved to the beginning of the discussion section. Starting with the interpretation of the current result consolidates a basis for continuing discussions. Maybe, the current first paragraph of the discussion section should be merged into the introduction section since it motivates the current research.**

As suggested, we have rewritten the Introduction and Discussion.

**Our responses to the comments of Reviewer #2**

**1. They found that SVH-1 overexpression was not able to rescue the regeneration defects in cdd-2 mutants, while SVH-2 overexpression was able to rescue. It’s not convincing that overexpression of SVH-2 was sufficient to transduce the signal to promote axon regeneration in the absence of ligand (non-protease variant of SVH-1), unless a constitutively active SVH-2 was used.**

We have previously shown that *svh-2* overexpression can suppress the *svh-1* defect (Ref. 5).

Polymerization of RTKs results in their constitutive activation. Thus, RTK overexpression induces their polymerization and activation.

**2. The authors argued that the timing of cdd-2 gene expression is critical for its role in editing svh-1 mRNA for promoting axon regeneration. In addition to showing cdd-2 expression pattern, this conclusion needs to be further confirmed by assessing axon regeneration in cdd-2 mutants at different stages (e.g. L3). If cdd-2 mutant shows no defects in axon regeneration at L3 stage, it would support that cdd-2 function in axon regeneration is time-specific. Also, time-specific transgenic expression of CDD-2 or SVH-1 variants through heat-shock promoter can help to further support their conclusion.**

Thompson et al. (Ref. 14) analyzed the expression pattern of *cdd-2* using RT-PCR. They reported that *cdd-2* was first expressed in L2 and increased in expression in young adults. Therefore, we predict that the *cdd-2* mutation is defective in axon regeneration at the L3 stage.

The question remains about why axons can regenerate at the L1 stage even if *cdd-2* is not expressed. Axon regeneration in *C. elegans* is regulated by multiple signaling pathways, such as JNK and p38 MAPK pathways. It is possible that the SVH-1–SVH-2 signaling pathway does not function at the L1 stage.

**3. Line 132, data on axon regeneration in ccd-1(ok390) are missing in Fig. 2C.**

We have presented data on axon regeneration in *cdd-1(ok390)* in Fig 2C.

**Our responses to the comments of Reviewer #3**

**1. To prove CDD-2 acts as cytosine nucleoside deaminase, it would be nice to test an enzyme-dead mutant of CDD-2 in axon regeneration.**

As suggested, we have examined an enzyme-dead mutant of CDD-2 in axon regeneration. The *ApoB* mRNA-editing activity of APOBEC-1 depends on its Cys-93 residue (Ref. 17). CDD-2 contains a conserved Cys-95 site that corresponds to mammalian Cys-93 (Fig 2A). We have created a *cdd-2(C95S)* mutant, in which Cys-95 is substituted with serine. The *cdd-2(C95S)* expression by the *srh-220* promoter in *cdd-2(km70)* mutants did not restore the axon regeneration defect (Fig 3C and S1 Table). Therefore, the enzymatic activity of CDD-2 is crucial for axon regeneration after laser ablation.

**2. Regarding the Asp-His-Ser catalytic triad, if all three amino acids must be present for SVH-1 protease activity, then any inactive SVH-1 mutation should rescue cdd-2 mutant phenotype. But in Figure 4B, H755Y and S898F behave differently. This argues that loss of protease activity is not sufficient for bypassing cdd-2. Biochemical experiments showing the amount of activity present in each mutant would be ideal (and also assessing binding to the receptor). Alternatively, the overall model should at least be revised.**

We agree with this comment. As suggested, we have revised the overall model. This comment is very helpful for the revision of our manuscript.

**3. Authors reported CDD-2 expression levels are low in L1 but increased in L4. So in L1, SVH-1 would mostly be protease-active form. However, L1 animals have been shown to regenerate well. This should be discussed.**

Axon regeneration in *C. elegans* is controlled by multiple signaling pathways, such as JNK and p38 MAPK pathways. The SVH-1–SVH-2 signaling pathway may not function at the L1 stage.

**4. Also regarding the timing of expression, the model predicts that overexpression CDD-2 under srh-220 promoter should inactive SVH-1 early in development, and cause developmental delay. It would be nice to test this.**

If *cdd-2* expression alone was sufficient to replace His-755 in *svh-1* mRNA with tyrosine, forced *cdd-2* expression at the L1 stage would cause larval arrest because the SVH-1 protein with Tyr-755 instead of His-755 loses protease activity. However, *cdd-2* overexpression from the *srh-220* promoter in wild-type animals did not affect larval development (S2B Fig), suggesting that expression of *cdd-2* alone is insufficient to induce the editing of *svh-1* mRNA. Further events, such as the activation of CDD-2 activity by axonal injury, may be necessary.

**Minor notes:**

**1. Lacking citations on line 82, line 119.**

We have presented these references.

**2. What is the nature of cdd-2(km70)? In method, the authors mentioned, “The strain obtained also contains an extra copy of the cdd-2 gene fragment, which carries a 3 bp in-flame 350 deletion”. Where’s the extra copy? Does it affect the interpretation of the mutant?**

The position of the extra copy on the chromosomes is currently unknown.

Based on the rescue of the *cdd-2(km70)* mutant phenotype by *cdd-2* expression (Fig 2C), the extra copy probably does not affect the interpretation of the mutant.

**3. Data on cdd-1(ok390) is mentioned on Fig 2C, but not presented.**

We have presented data on axon regeneration in *cdd-1(ok390)* in Fig 2C.

**4. Data presented Figure 3B, Psrh-220::svh-1 group, did not agree with data from Figure S1 table.**

We have corrected these data in S1 Table.
