# Supplementary material for: A cytidine deaminase regulates axon regeneration by modulating the functions of the Caenorhabditis elegans HGF/plasminogen family protein SVH-1
Source: PLoS Genet. 2024 Jul 26;20(7):e1011367. doi: 10.1371/journal.pgen.1011367 (PMC11305532; doi:10.1371/journal.pgen.1011367)
Supplement: Attachment — Submitted filename: revise-response letter.docx [file pgen.1011367.s009.docx]

Dear Drs. Hart and Dr. Copenhaver,

Please find enclosed our revised manuscript entitled, “A cytidine deaminase regulates axon regeneration by modulating the functions of the *Caenorhabditis elegans* HGF/plasminogen family protein SVH-1” (by Shimizu et al., PGENETICS-D-23-01297). We would like to thank you for your e-mail of 20 May 2024, as well as the reviewers’ comments on our manuscript. We appreciate these comments, which have enabled us to improve the manuscript. We have conducted further experiments that have been designed to address the comments of the reviewers. We hope this revised manuscript is now acceptable for publication in *PLOS Genetics.* Our point-by-point responses to the reviewers’ comments are provided below.

With best regards,

Kunihiro Matsumoto

Paper: PGENETICS-D-23-01297

Authors: Shimizu et al.,

Title: A cytidine deaminase regulates axon regeneration by modulating the functions of the *Caenorhabditis elegans* HGF/plasminogen family protein SVH-1

The manuscript has been revised according to the comments of the Reviewing Editor and the three reviewers. Our responses to the comments are as follows:

**Responses to the comments of the Reviewing Editor**

**Reviewer #1: The authors' responses mostly satisfied the concerns I suggested. The only two things that I strongly suggest improving are:**

**1. The manuscript still omits the description of the limitations of the current study and sometimes it makes the audience skeptical. The previous comment No. 4 pointed out the question about how spatial signal transportation occurs and it would be beneficial to discuss about this in the discussion section. Also, related to the previous comment No.5, the CENGEN results show the expression of svh-1 in the other neurons. Together with the fact that ref.5 only uses the NLS-fused expression of svh-1, a clear description of the intention of the experiment and the limitation of the design should make a solid story.**

As suggested, we have discussed these points in the discussion section.

**2. Related to the previous comment No. 6, it is highly suggested to consult with a good statistician. Clearly, the problem of the multi-comparison in this study is not the case in Ref. 30. The paper discusses the different layers of the problem that are clearly shown in the title. And if we closely look into the reference about the adjustment in multiple-testing as shown in Ref.30 (the following paper), this is a case study of the clinical research and no theoretical validation is described.
10. Wason J.M.S., Stecher L., Mander A.P. Correcting for multiple-testing in multi-arm trials: is it necessary and is it done? Trials. 2014;15(1):364.
The experiments of mutant and rescue lines are highly related and can not be regarded as distinct tests. Moreover, the main point of the previous comment was the proper use of Fisher's exact test. It is still doubtful to be inappropriate use of the method.**

As suggested, we have employed the Chi-square test instead of the Fisher's exact test, incorporated additional regeneration data, and conducted multi-comparison with the FDR correction applied as necessary.
